# Should we treat sepsis-induced DIC with anticoagulants?

**DOI:** 10.1186/s40560-020-0435-8

**Published:** 2020-02-14

**Authors:** Yu Inata

**Affiliations:** Department of Intensive Care Medicine, Osaka Women’s and Children’s Hospital, 840 Murodo-cho, Izumi, Osaka 594-1101 Japan

**Keywords:** Disseminated intravascular coagulation, Sepsis, Anticoagulation, Immunothrombosis, Precision medicine

## Abstract

**Background:**

Disseminated intravascular coagulation (DIC) is a common complication in sepsis because of crosstalk between the immune system and the coagulation system. Several anticoagulant agents have been tested in an attempt to improve the survival of patients with sepsis and sepsis-induced DIC. Here, we discuss the rationale against using anticoagulation therapy in septic DIC.

**Main body of the abstract:**

Coagulopathy and DIC are associated with increased mortality in sepsis. Several anticoagulant agents have been tested in an attempt to improve the survival of patients with sepsis and sepsis-induced DIC, but have proven largely ineffective. This is because of two major factors. First, the coagulation system is complex and closely related to the immune system. When we manipulate one of the factors involved in these systems, we may disturb the delicate homeostasis between them. A second factor may be failure to identify patients who will benefit from anticoagulation therapy. This may be attributed partly to the fact that there is no gold standard for the diagnosis of DIC, and there are consequently several diagnostic criteria, none of which are specifically designed for sepsis-induced DIC. Application of precision medicine, of the kind currently being applied in other intensive care fields, may be the key to overcoming these challenges. Until we know the precise target population, we should not use anticoagulation therapy in sepsis-induced DIC outside a research setting.

**Short conclusion:**

There is no strong evidence to support the effectiveness of routine anticoagulation therapy in sepsis-induced DIC, and it should not be used clinically until more is known regarding the population of patients who may benefit from it.

## Background

Disseminated intravascular coagulation (DIC) is an acquired syndrome characterized by delocalized intravascular activation of coagulation arising from a range of different causes [1]. If sufficiently severe, it can lead to organ dysfunction and death. DIC is frequently seen in sepsis because the immune system and coagulation system closely interact with each other [2]. Coagulopathy and DIC are associated with increased mortality in sepsis [3, 4]. Therefore, several anticoagulant agents have been tested in an attempt to improve the survival of patients with sepsis and sepsis-induced DIC. Here, rationales against using anticoagulation therapy in septic DIC are discussed.

## Main text

Observational studies suggest a link between the severity of coagulopathy, organ dysfunction, and death in patients with sepsis [3, 4]. Therefore, it is not surprising that several anticoagulant agents have been tested in an attempt to improve the survival of patients with sepsis and sepsis-induced DIC. Such attempts have been unsuccessful in improving survival in sepsis and septic shock [5, 6]. By network meta-analysis, Yatabe et al. showed no significant differences in mortality risk when a placebo and four anticoagulants (antithrombin, thrombomodulin, heparin, or protease inhibitors) were compared in patients with sepsis-induced DIC [6]. Recently, Vincent et al. reported the results of the SCARLET (Sepsis Coagulopathy Asahi Recombinant LE Thrombomodulin) phase 3 trial [7]. The inclusion criteria of the SCARLET study were based on a post hoc analysis of a randomized placebo-controlled phase 2b study, in which recombinant human soluble thrombomodulin (rhsTM) had a non-significant effect on the 28-day mortality rate in patients with sepsis and suspected DIC (17.8% vs 21.6% in the placebo group) [8]. Despite including only patients with sepsis-associated coagulopathy, the SCARLET trial failed to demonstrate a reduction in 28-day all-cause mortality. Incorporating then-unpublished results of the SCARLET trial, Yamakawa et al. conducted a systematic review and meta-analysis of the effects of rhsTM on sepsis-induced coagulopathy [9]; reduction in the risk of all-cause 28-day mortality in the rhsTM group was not significantly different compared to the control group (relative risk, 0.87; 95% confidence interval, 0.74–1.03; *p* = 0.10).

Why have anticoagulants failed to demonstrate survival benefit in sepsis or sepsis-induced DIC? Firstly, the coagulation system, traditionally divided into an intrinsic and extrinsic pathway, is not as simple as once thought and closely related to the immune system [10]. Immunothrombosis is an evolutionarily conserved mechanism in which thrombosis plays a major physiological role in immune defense [11]. When we manipulate factors involved in these intertwined systems, we may disturb their delicate homeostasis unless we understand and control for all unintended effects. We are all too familiar with the fact that many clinical trials involving strategies for modifying the systemic inflammatory response by targeting endogenous mediator molecules have produced negative results [12]. Secondly, there has been a failure to identify patients who would benefit from anticoagulation therapy. The disappointing results of the SCARLET trial could be explained by the fact that sepsis-associated coagulopathy is not the same as sepsis-induced DIC; coagulopathy is common in sepsis but does not necessarily lead to the development of a widespread coagulopathy that meets the diagnostic criteria of DIC [3]. A meta-analysis of randomized controlled trials of anticoagulant therapies in sepsis demonstrated a survival benefit with anticoagulation only in the subgroup of patients with sepsis-induced DIC, but not in the population with sepsis-associated coagulopathy [5]. In the editorial accompanying the SCARLET trial report [13], it is noted that failure to identify patients who were more likely to respond favorably to rhsTM could partially explain the lack of efficacy for rhsTM in reducing mortality. However, this does not mean that rhsTM is proven to be effective in reducing mortality when used in patients with confirmed sepsis-induced DIC, and physicians should be reminded of the difficulty of identifying patients who may benefit from rhsTM. Difficulty identifying the right targets for anticoagulation therapy can be attributed partly to the ambiguous diagnostic criteria for DIC. There is no gold standard for DIC diagnosis, and there are several diagnostic criteria; in addition, none of these criteria were specifically designed for sepsis-induced DIC [4]. Thus, discrimination between simple coagulopathy and DIC and the timing of treatment becomes somewhat arbitrary, and the optimal cutoff points of DIC scoring systems are still under debate [14]. Finally, different sepsis phenotypes correlated with host-response patterns and clinical outcomes may explain the heterogeneity of treatment effects [15].

How can we overcome the challenges mentioned above? One solution could be the application of precision medicine, which is increasingly common in the field of intensive care medicine [16]. One example is the application of prognostic and predictive enrichment strategies to find populations that could benefit from corticosteroid treatments in sepsis [17]. Predictive enrichment strategy involves identifying endotypes (subclasses of a disease or syndrome as defined by function or biology) by analyzing gene expression [18]. This highly sophisticated method has revealed that the endotype of patients with sepsis changes over time, possibly affecting the response of these patients to treatment [19]. If this dynamic change of endotype is seen in patients with septic DIC, it may not be beneficial to administer rhsTM to all patients with septic DIC for a uniform duration.

There is hope in the field of DIC. In patients with sepsis, thrombocytopenia on admission was associated with a distinct gene expression pattern that corresponds to a more disturbed host response [20]. Applying a precision medicine approach to patients with sepsis-induced DIC throughout the clinical course may help identify patients who benefit from anticoagulation. Until such measures become available, the best possible targets for anticoagulation therapy may be patients fulfilling all three criteria of sepsis, DIC, and high disease severity [21]. Nevertheless, in the absence of robust evidence, anticoagulants should only be used for sepsis-induced DIC in a research setting in order to try and find the right population, timing, dosage, and duration of therapy for effective treatment.

## Conclusions

There is no strong evidence to support routine anticoagulation therapy in sepsis-induced DIC. While anticoagulation therapy may be beneficial for some patients, there are several challenges that need to be overcome in order to find the right targets of the therapy. Until we know the precise target population, we should not use anticoagulation therapy in sepsis-induced DIC outside a research setting.

## Data Availability

Not applicable

## Abbreviations

DIC: Disseminated intravascular coagulation
rhsTM: Recombinant human soluble thrombomodulin
SCARLET: Sepsis Coagulopathy Asahi Recombinant LE Thrombomodulin

## References

[1] Taylor Fletcher, Toh Cheng-Hock, Hoots Keith, Wada Hideo, Levi Marcel (2001). Towards Definition, Clinical and Laboratory Criteria, and a Scoring System for Disseminated Intravascular Coagulation. Thrombosis and Haemostasis.

[2] Levi M, van der Poll T (2017). Coagulation and sepsis. Thromb Res.

[3] Lyons PG, Micek ST, Hampton N, Kollef MH (2018). Sepsis-associated coagulopathy severity predicts hospital mortality. Crit Care Med.

[4] Iba T, Di Nisio M, Levy JH, Kitamura N, Thachil J (2017). New criteria for sepsis-induced coagulopathy (SIC) following the revised sepsis definition: a retrospective analysis of a nationwide survey. BMJ Open.

[5] Umemura Y, Yamakawa K, Ogura H, Yuhara H, Fujimi S (2016). Efficacy and safety of anticoagulant therapy in three specific populations with sepsis: a meta-analysis of randomized controlled trials. J Thromb Haemost.

[6] Yatabe T, Inoue S, Sakamoto S, Sumi Y, Nishida O, Hayashida K (2018). The anticoagulant treatment for sepsis induced disseminated intravascular coagulation; network meta-analysis. Thromb Res.

[7] Vincent JL, Francois B, Zabolotskikh I, Daga MK, Lascarrou JB, Kirov MY (2019). Effect of a recombinant human soluble thrombomodulin on mortality in patients with sepsis-associated coagulopathy: the SCARLET randomized clinical trial. JAMA..

[8] Vincent JL, Ramesh MK, Ernest D, Larosa SP, Pachl J, Aikawa N (2013). A randomized, double-blind, placebo-controlled, phase 2b study to evaluate the safety and efficacy of recombinant human soluble thrombomodulin, ART-123, in patients with sepsis and suspected disseminated intravascular coagulation. Crit Care Med.

[9] Yamakawa K, Murao S, Aihara M (2019). Recombinant human soluble thrombomodulin in sepsis-induced coagulopathy: an updated systematic review and meta-analysis. Thromb Haemost.

[10] Antoniak S (2018). The coagulation system in host defense. Res Pract Thromb Haemost.

[11] Engelmann B, Massberg S (2013). Thrombosis as an intravascular effector of innate immunity. Nat Rev Immunol.

[12] Marshall JC (2014). Why have clinical trials in sepsis failed ?. Trends Mol Med.

[13] van der Poll T (2019). Recombinant human soluble thrombomodulin in patients with sepsis-associated coagulopathy: another negative sepsis trial?. JAMA..

[14] Yamakawa K, Umemura Y, Murao S, Hayakawa M, Fujimi S (2019). Optimal timing and early intervention with anticoagulant therapy for sepsis-induced disseminated intravascular coagulation. Clin Appl Thromb Hemost.

[15] Seymour CW, Kennedy JN, Wang S, Chang CH, Elliott CF, Xu Z (2019). Derivation, validation, and potential treatment implications of novel clinical phenotypes for sepsis. JAMA..

[16] Wong HR (2017). Intensive care medicine in 2050: precision medicine. Intensive Care Med.

[17] Wong HR, Atkinson SJ, Cvijanovich NZ, Anas N, Allen GL, Thomas NJ (2016). Combining prognostic and predictive enrichment strategies to identify children with septic shock responsive to corticosteroids. Crit Care Med.

[18] Scicluna BP, van Vught LA, Zwinderman AH, Wiewel MA, Davenport EE, Burnham KL (2017). Classification of patients with sepsis according to blood genomic endotype: a prospective cohort study. Lancet Respir Med.

[19] Wong HR, Cvijanovich NZ, Anas N, Allen GL, Thomas NJ, Bigham MT (2018). Endotype transitions during the acute phase of pediatric septic shock reflect changing risk and treatment response. Crit Care Med.

[20] Claushuis TA, van Vught LA, Scicluna BP, Wiewel MA, Klein Klouwenberg PM, Hoogendijk AJ (2016). Thrombocytopenia is associated with a dysregulated host response in critically ill sepsis patients. Blood..

[21] Umemura Y, Yamakawa K (2018). Optimal patient selection for anticoagulant therapy in sepsis: an evidence-based proposal from Japan. J Thromb Haemost.

